# Stable Isotope Marking of Laboratory-Reared *Aedes aegypti* (Diptera: Culicidae)

**DOI:** 10.1093/jme/tjz210

**Published:** 2019-11-21

**Authors:** Selene M Garcia-Luna, Jose G Juarez, Sofia Cabañas, Wendy Tang, E Brendan Roark, Christopher R Maupin, Ismael E Badillo-Vargas, Gabriel L Hamer

**Affiliations:** 1 Department of Entomology, Texas A&M University, College Station, TX; 2 Departamento de Biologia, Universidad del Valle de Guatemala, Guatemala; 3 Stable Isotope Geosciences Facility, Department of Geography, Texas A&M University, College Station, TX; 4 Department of Entomology, Texas A&M AgriLife Research, Weslaco, TX

**Keywords:** *Aedes aegypti*, stable isotope, marker retention, mark-capture study, dispersal

## Abstract

The use of stable isotope enrichment to mark mosquitoes has provided a tool to study the biology of vector species. In this study, we evaluated isotopic marking of *Aedes aegypti* (L.) (Diptera: Culicidae) in a laboratory setting. We determined the optimal dosage for marking adult *Ae. aegypti* mosquitoes with ^13^C and ^15^N. Additionally, *Ae. aegypti* mosquitoes were single and dually marked with ^13^C and ^15^N for up to 60 d postemergence without changes to adult body size or transgenerational marking. This report adds to the growing literature that explores the use of alternative marking methods for ecological and vector biology studies.

*Aedes aegypti* (L.) (Diptera: Culicidae) has spread throughout tropical and semitropical regions of the world, has a close association with humans, and has become a primary vector of viruses including dengue (DENV), chikungunya (CHIKV), Zika (ZIKV), and yellow fever (YFV) (Powell 2018). With no efficacious vaccines available for DENV, CHIKV, and ZIKV (Espinal et al. 2019), vector control remains a primary strategy to reduce vector populations and transmission of viruses to humans (Barrera et al. 2014, Schwab et al. 2018). In order to develop strategies and devices to control disease vectors such as *Ae. aegypti,* there is a need to improve the tools to study their biology.

The development of a marking technique is crucial for the study of vector biology such as feeding habits, resource allocation, and dispersal studies (Faiman et al. 2019). The use of stable isotopes as a biological marker has been documented for several aquatic insects including mosquitoes of the genera *Anopheles* (Diptera: Culicidae) (Hood-Nowotny et al. 2006, Helinski et al. 2007, Opiyo et al. 2016), *Culex* (Diptera: Culicidae) (Hamer et al. 2012, Winters and Yee 2012, Hamer et al. 2014, Medeiros et al. 2017), and *Aedes* (Winters and Yee 2012, Opiyo et al. 2016, Medeiros et al. 2017). However, there has not been a published use of isotopes to mark *Ae. aegypti* mosquitoes. In this study, we document the use of the stable isotopes ^13^C and ^15^N to isotopically mark laboratory-reared *Ae. aegypti* mosquitoes.

## Materials and Methods

We used *Ae. aegypti* mosquitoes from the Liverpool strain to evaluate the effects of single and dual isotopic enrichment of a hay-infusion larval habitat on male and female adults.

### Isotopic Enrichment of Larval Habitat

In order to simulate a breeding site, plastic trays (34.3 × 25.4 × 3.8 cm of 3 liters) were prepared 1 wk prior to eclosion of mosquito eggs by adding 1 g of hay/liter of water, 0.002 g/liter of ^13^C and/or ^15^N (Hamer et al. 2012). Initial experiments combining isotopes and eggs on the same day yielded low enrichment (S. M. Garcia-Luna, J. G. Juarez and G. L. Hamer, unpublished data). Trays were placed into an environmental chamber set up to 28°C and a photoperiod of 12:12 light–dark hours to allow for microbial communities to develop. Two-hundred eggs per tray were added and followed until pupation, with the exception of the dose experiment on which 50 eggs per dose were used. When larvae reached L3, an additional 0.5 g of grounded fish food (TetraMin, Tetra, Germany) was added per tray as a nutrition supplement. Pupae were collected and placed in 100 ml plastic cups inside a BugDorm-1 (MegaView Science Co., Ltd., Taiwan). Adults were offered 10% sucrose solution ad libitum, which was changed weekly. Three individuals per treatment by experiment were collected into individual 1.7 ml microcentrifuge tubes and stored at −80°C until further processing. Control samples were reared as previously described without the addition of stable isotopes.

### Single and Dual Isotope Marking

Mosquitoes were reared in either 0.002 g/liter of ^13^C-labeled glucose (U-^13^C_6_, 99% atom%; Cambridge Isotope Laboratories, Inc., Andover, MA), 0.002 g/liter of ^15^N-labeled potassium nitrate (KNO_3_, ^15^N, 99% atom%; Cambridge Isotope Laboratories, Inc.) (Hamer et al. 2012) or a combination of both for the dual isotopic label (1:1 ratio) treatment. We tested if mosquito sex (male and female) and the time after adult emergence had an effect on the δ ^13^C and δ ^15^N by sampling adult mosquitoes at 7, 14, 21, 32, 39, and 60 d postemergence (dpe). We also evaluated three isotopic doses of ^13^C by hatching and rearing *Ae. aegypti* mosquitoes at concentrations of 0.001, 0.00075, and 0.00035 g/liter. We assessed the potential for transgenerational marking by allowing 3–5-d-old ^13^C-marked male (*n* = 50) and female (*n* = 50) mosquitoes to mate and produce offspring that were tested upon adult emergence.

In order to determine the effects of stable isotope enrichment on adult male body size, wing length measurements (*n* = 15 males/treatment) were taken. Subsequently, we evaluated if the addition of 400 µl of an artificial diet made of a 2% solution of desiccated and defatted liver powder (Bio-Serv, Flemington, NJ) and brewer’s yeast hydrolysate (Bio-Serv) at a ratio of 3:2 (which we will refer as LP) during egg eclosion, would influence δ ^13^C and δ ^15^N.

### Sample Processing and Analysis

Samples were analyzed for isotopic marking at the Texas A&M University Stable Isotope Geosciences Facility using a Thermo Fisher Scientific Delta V Advantage with Flash EA Isolink attached to a ThermoFinnigan Conflo IV isotope ratio mass spectrometer as previously described (McDermott et al. 2019). Individual adult mosquitoes were placed in tin capsules in a 96-well plate. Mosquitoes were dried at 50°C for 24 h, and capsules were then crimped shut (Medeiros et al. 2017). δ ^13^C (measurement of the ratio of stable isotopes ^13^C: ^12^C) versus Vienna Pee Dee Belemnite (VPDB) and δ ^15^N (measurement of the ratio of stable isotopes ^15^N: ^14^N) versus air ([(R_sample_-R_standard_)/R_standard_] × 1,000) (IAEA 1995) values for each pool were recorded, regardless of isotope treatment. Differences in the mean δ ^13^C or δ ^15^N by treatment were analyzed using a two-sample *t*-test. Wing length was analyzed by using a nonparametric comparison (due to non-normality of data) using Dunn’s test with control for joint ranking. Normality was evaluated using the Shapiro Wilk test on the residuals.

## Results and Discussion

**Fig. 1.**
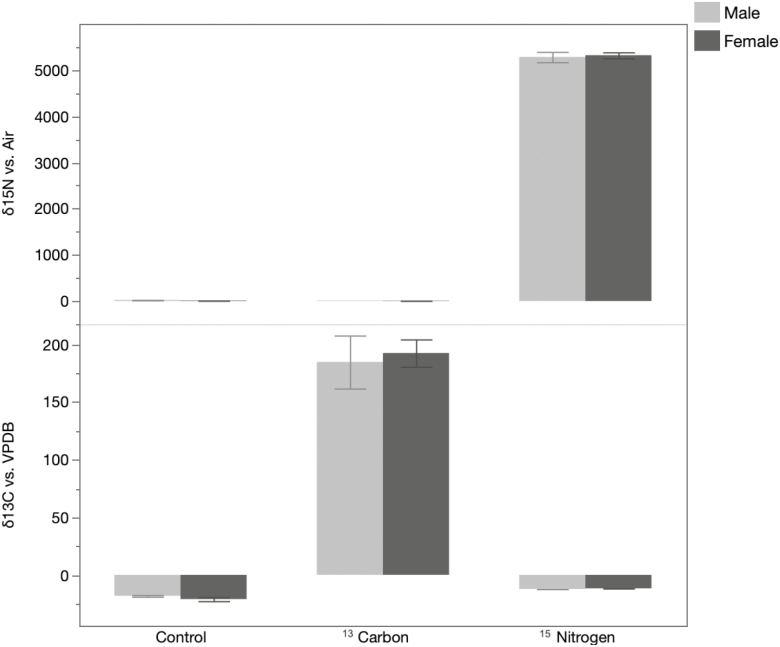
Single isotopic marking for δ ^15^N and δ ^13^C in adult male and female *Ae. aegypti* 24 h postemergence. Light gray bars denote *Ae. aegypti* males and dark gray *Ae. aegypti* females. Error bars indicate SEM.

**Fig. 2.**
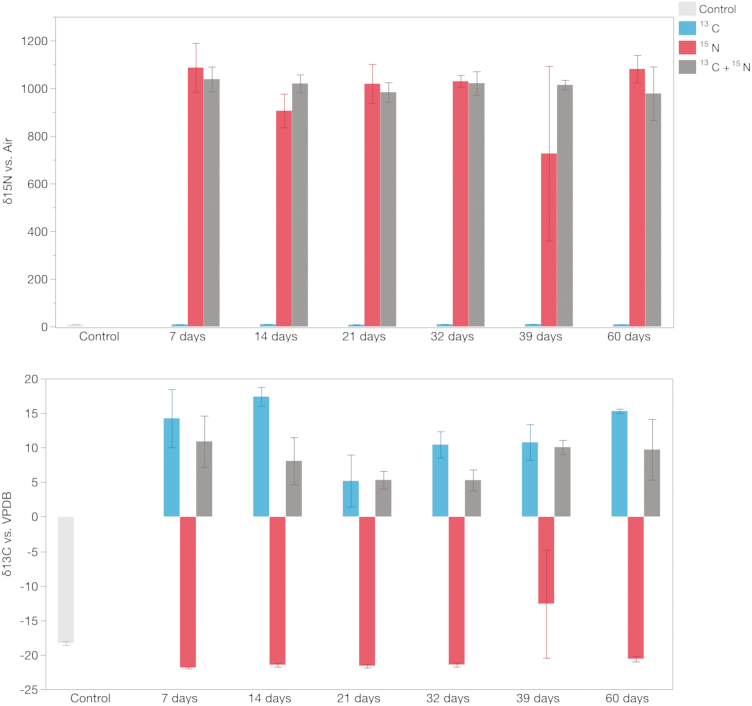
Persistence of single (^13^C, ^15^N) and dual (^13^C+ ^15^N) isotopic marking over time. Upper panel shows the δ values for ^15^N (ratio of stable isotopes ^15^N: ^14^N) and lower panel the δ values for ^13^C (ratio of stable isotopes ^13^C: ^12^C) (difference in scales is due to a higher ratio found in δ ^15^N). Light gray bars indicate non-marked (control) individuals. Single isotopic marking with nitrogen (^15^N) is represented with red bars, carbon (^13^C) with blue bars and dual isotopic marking (^13^C+ ^15^N) with dark gray bars. Error bars indicate SEM.

We did not observe a difference in the δ values between male and female *Ae. aegypti* for either δ ^13^C (m = 185, f = 193, *t* = 0.29, *P* = 0.7) or δ ^15^N (m = 5284, f = 5322, *t* = 0.29, *P* = 0.7; Fig. 1). The persistence of single and dual marking over time was evaluated up to 60 dpe. The δ-values showed that single (δ ^13^C: dpe 60 = 15.31 ± 0.31; δ ^15^N: dpe 60 = 1081.41 ± 100.30) and dual (δ ^13^C: dpe 60 = 9.05 ± 4.54; δ ^15^N: dpe 60 = 996.93 ± 116.61) marking persist over time, at least under our laboratory experimental setting, and that it can be effectively measured if single or dual marking is carried out (Fig. 2). We did not detect a statistical difference when comparing the mean concentration of single or dual marking throughout the study, with the exception of δ ^13^C for 14 (^13^C = 17.4, ^13^C+ ^15^N = 8.08, *t* = −3.1, *P* = 0.02), 32 (^13^C = 10.4, ^13^C+ ^15^N = 5.3, *t* = −2.57, *P* = 0.04) and 60 d postadult emergence (^13^C = 15.3, ^13^C+ ^15^N = 9.1, *t* = −2.8, *P* = 0.03). This reduction for δ ^13^C during the dual marking could be related to differential assimilation of carbon and nitrogen under different relative abundances of these elements. For example, in the presence of elevated nitrogen under the dual marking treatment, the larvae or larval diet could have assimilated less carbon.

**Fig. 3.**
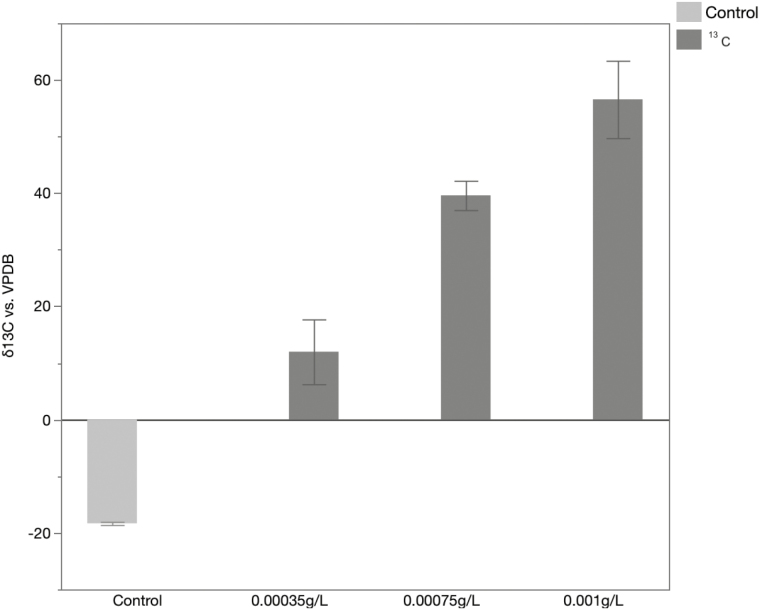
Dosage experiment with ^13^C. Light gray bars indicate nonenriched (control) individuals. Dark gray bars represent *Ae. aegypti* individuals enriched at increasing concentrations of ^13^C. Error bars indicate SEM.

When evaluating the three concentrations of ^13^C, we observed a significantly higher δ ^13^C in comparison to the control (−18.32 ± 0.49; Fig. 3). This confirms that even at the lower tested dose (0.00035 g/liter) an effective marking can be detected (11.99 ± 9.92), allowing for lower concentrations to be used for enrichment purposes. To evaluate the potential for transgenerational marking, adult females and males with an average δ ^13^C of 10.91 ± 7.9 produced progeny with values of −16.62 ± 0.29 for females and −17.60 ± 0.28 for males, demonstrating no transfer of ^13^C marking to F1 eggs (data not shown).

We assessed the effect of single and dual larval diet isotope enrichment on the wing length (mm) of adult male *Ae. aegypti*. Males that had dual marking showed a statistically shorter wing length than the control males (control = 2.24 ± 0.19, dual = 2.08 ± 0.07, Z = −3.26, *P* = 0.003). When comparing the control to single isotopic marking with ^13^C (2.14 ± 0.05mm) and ^15^N (2.30 ± 0.14) no statistical difference was observed. However, wing length of our artificially fed males were all lower than the average 2.64 mm of field collected ones (Nasci 1986). This suggest that the larval environment relying on the natural microbiota provided by the hay infusion and the addition of fish food at the L3 stage provided insufficient nutrition for developing larvae. In light of this, we evaluated the addition of the artificial diet as supplemental nutrition following the previous procedures. We were able to detect the isotopic marking of males (δ ^13^C: 389.98 ± 15.01; δ ^15^N: 5,531.84 ± 70.33) and females (δ ^15^N: 5,694.16 ± 164.62) when artificial food was added. We were unable to collect sufficient females for the ^13^C isotopic marking. This demonstrates that the early addition of artificial food for nutrition allowed the bioaccumulation of stable isotopes into adult structural tissues to persist.

This study documents that similar protocols to isotopically mark *Culex*, *Anopheles*, and other species of *Aedes* mosquitoes also apply to *Ae. aegypti*. This study extends prior laboratory marking studies by showing very little decay over the 60-d period as adults (Hamer et al. 2012). The lack of transgenerational marking of progeny improves the utility of using stable isotopes in a mark-capture study design. As a container species mosquito and as a vector responsible for 96 million cases of dengue each year (Bhatt et al. 2013), the use of stable isotope marking offers a valuable tool to study the bionomics of *Ae. aegypti*.
